# ﻿A new genus and two new species of Wiesneriomycetaceae (Tubeufiales) from China revealed by molecular phylogeny and taxonomy

**DOI:** 10.3897/mycokeys.123.167204

**Published:** 2025-09-26

**Authors:** Heng Pan, Kai-Rong Wang, Ming-Yi Zhang, Xiao-Kang Ren, Bing-Da Sun, Gang Tao, Zhi-Yuan Zhang

**Affiliations:** 1 College of Eco-Environmental Engineering, Guizhou Minzu University, Guiyang 550025, China Guizhou Minzu University Guiyang China; 2 China General Microbiological Culture Collection Center, Institute of Microbiology, Chinese Academy of Sciences, Beijing 100101, China Institute of Microbiology, Chinese Academy of Sciences Beijing China

**Keywords:** *
Chlamydosporoides
*, epiphytic soil, new taxa, taxonomy, tree holes

## Abstract

While investigating culturable mycobiota in epiphytic soils found in urban tree holes from Guizhou Province, China, a new genus along with two new species of the family Wiesneriomycetaceae were discovered based on a combination of morphological characteristics, molecular evidence, and physiological features. Phylogenetic analyses of SSU, ITS, LSU, and *tef1* sequences indicate that our new collections form a distinct clade; thus, *Chlamydosporoides***gen. nov.** is proposed. This genus is distinguished from other genera in Wiesneriomycetaceae by the absence of acropetal conidial chains, setae, synnemata, sporodochia, or stromata. We describe and illustrate the new genus *Chlamydosporoides* and the new species (*C. guizhouensis***sp. nov.** and *C. sinensis***sp. nov.**) herein, and we discuss their phenotypic and genotypic differences from allied genera.

## ﻿Introduction

Suetrong et al. (2014) introduced the family Wiesneriomycetaceae to accommodate *Wiesneriomycetales conjuntosporus* and *W. laurinus*, forming a sister lineage to Tubeufiales. Bezerra et al. (2017) subsequently established this genus, *Wiesneriomycetales*, to accommodate this family. However, Wijayawardene et al. (2020) and Hongsanan et al. (2020) reclassified *Wiesneriomycetales* as a synonym of Tubeufiales and placed Wiesneriomycetaceae within the latter. The family Wiesneriomycetaceae is characterized by sporodochial conidiomata with subulate setae, macronematous conidiophores, and acropetal conidial chains connected by a narrow isthmus (Suetrong et al. 2014; Bezerra et al. 2017). According to Wijayawardene et al. (2020), Wiesneriomycetaceae comprises six genera: *Parawiesneriomyces*, *Phalangispora*, *Pseudogliophragma*, *Setosynnema*, *Speiropsis*, and *Wiesneriomyces*. Later, Xu et al. (2022) established the genus *Heveicola* (with *H. xishuangbannaensis* as its type species) within Wiesneriomycetaceae and provided a key to the genera of this family. Hyde et al. (2024) also included *Excipulariopsis*, bringing the total to eight genera, though this conclusion was not based on data analysis. Members of Wiesneriomycetaceae are widely distributed in tropical and subtropical regions and colonize diverse substrates, including decaying twigs, leaves, and soil (Suetrong et al. 2014; Pratibha et al. 2015; Crous et al. 2016).

Tree holes are important structural units in forest ecosystems and a key component of the forest canopy. They can be broadly categorized into two types: (1) cavities excavated by birds or insects, which primarily serve as shelters or habitats for animals; (2) natural hollows formed in living trees. When large branches break or tree trunks become damaged, fungi, bacteria, and insects invade the bark and sapwood, leading to the gradual formation of tree holes. Once formed, these cavities collect rainwater and organic debris (e.g., fallen leaves and twigs), becoming phytotelmata that serve as microhabitats for small arthropods, as well as important substrates for epiphytic plants, parasitic plants, and microorganisms (Basset 2001; Ellwood and Foster 2004; Majumdar et al. 2012). As a unique microhabitat in terrestrial ecosystems, tree holes harbor distinctive fungal communities. Studies have revealed that the fungal diversity in epiphytic soils within tree holes is lower than the diversity in ground soils, with significantly different community compositions between these two habitats (Zheng et al. 2017).

In 2024, during our investigation of fungal communities in epiphytic soils of urban tree holes in Guizhou Province, China, seven isolates belonging to Wiesneriomycetaceae were obtained. This study aims to: 1) describe a new genus, *Chlamydosporoides* gen. nov., along with two new species; 2) provide a checklist that includes substrate, molecular data availability, morphological characteristics and country of origin.

## ﻿Materials and methods

### ﻿Fungal isolation and morphology

Soil samples were collected from tree holes at two locations in Huaxi District, Guiyang, Guizhou Province: (1) *Camphora officinarum* Boerh.ex Fabr. at Guizhou Minzu University (25 July 2024), (2) *Catalpa ovata* G. Don at Guizhou University South Campus (28 September 2024). Samples were collected and stored in sterile plastic bags, transported to the laboratory under refrigeration (4 °C), and processed immediately upon arrival. Fungal isolates were obtained and purified following the methods described by Zhang et al. (Zhang et al. 2023, 2024). Briefly, 2 g of each sample were suspended in 20 ml of sterile water in a 50 ml sterile conical flask. The conical flasks were thoroughly shaken using a Vortex vibration meter. The suspension was then diluted to a concentration of 10^-4^. Then, 1 ml of the diluted sample was transferred to a sterile Petri dish, and Martin’s medium (KH_2_PO_4_ 1 g/L, MgSO_4_ 0.5 g/L, peptone 5 g/L, glucose 2 g/L, agar 20 g/L, 1% Bengal red aqueous solution 3.3 mL/L), Sabouraud’s dextrose agar (SDA; peptone 10 g/L, dextrose 40 g/L, agar 20 g/L, 1% Bengal red aqueous solution 3.3 mL/L), and Sabouraud’s dextrose agar yeast extract (SDAY; peptone 10 g/L, dextrose 40 g/L, agar 20 g/L, yeast extract 2 g/L, 1% Bengal red aqueous solution 3.3 mL/L) containing 50 mg/L penicillin and 50 mg/L streptomycin, was added and mixed. Three replicates of each medium were made. Plates were incubated at 25 °C for 1–2 weeks, and single colonies were selected from the plates and inoculated into new potato dextrose agar (PDA, potato 200 g/L, dextrose 20 g/L, agar 20 g/L) plates.

Pure colonies isolated on PDA medium were subcultured onto fresh PDA, Oatmeal Agar (OA), and Synthetic Nutrient-poor Agar (SNA) plates, and incubated at 25 °C in darkness for 14 days to assess colony morphology and macroscopic characteristics. Following the methodology of Guo et al. (2019), isolates were inoculated onto Corn Meal Agar (CMA) plates and incubated at 28 °C in darkness for 1 week to examine setae formation, conidiomata development, and sexual morph production. Morphological features were observed and recorded using a Zeiss Axio Imager A2 microscope, and a Zeiss AxioCam MRc color digital camera (Carl Zeiss Ltd., Munchen, Germany). The holotype (dried at 50 °C) of the novel species was deposited at the Fungarium (HMAS), Institute of Microbiology, Chinese Academy of Sciences (CAS). The ex-type living cultures of the novel taxa were preserved in the China General Microbiological Culture Collection Center (CGMCC). All living cultures were cryopreserved in sterile 30% glycerol at -80 °C and deposited at the College of Eco-Environmental Engineering, Guizhou Minzu University. The taxonomic descriptions of the new taxa were registered in MycoBank.

### ﻿DNA extraction, PCR amplification and sequencing

The BioTeke Fungal Genomic DNA Extraction Kit (DP2032, BioTeke, Beijing, China) was used to extract genomic DNA from fungal hyphae according to the manufacturer’s instructions. Four gene regions (SSU, ITS, LSU, and *tef1*) were amplified and sequenced using primers listed in Table 1. The 25 μL PCR mixture contained: 2.0 μL DNA template, 1.0 μL each of forward and reverse primers, 8.5 μL ddH_2_O, and 12.5 μL 2× MasterMix (Sangon Biotech, China). All newly generated sequences were deposited in GenBank (Table 2).

**Table 1.** Sequences of primers used in this study.

Molecular marker	Primer name (forward/reverse)	Primer sequence (5´-3´)	Reference
SSU	NS1	GTAGTCATATGCTTGTCTC	White et al. (1990)
	NS4	CTTCCGTCAATTCCTTTAAG	White et al. (1990)
ITS	ITS1	TCCGTAGGTGAACCTGCG	White et al. (1990)
	ITS4	TCCTCCGCTTATTGATATGC	White et al. (1990)
LSU	LR0R	ACCCGCTGAACTTAAGC	Vilgalys and Hester (1990)
	LR5	ATCCTGAGGGAAACTTC	Vilgalys and Hester (1990)
*tef1*	983F	GCYCCYGGHCAYCGTGAYTTYAT	Rehner and Buckley (2005)
	2218R	ATGACACCRACRGCRACRGTYTG	Rehner and Buckley (2005)

**Table 2.** Strains used in this study, with information on the GenBank accessions of the sequences.

Species	Strains	GenBank accession no.			
		SSU	ITS	LSU	*tef1*
*Acanthostigma chiangmaiense* Boonmee & K.D. Hyde	MFLUCC 10-0125 T	JN865185	JN865209	JN865197	KF301560
*Allophaeosphaeria muriformia* (Ariy., Camporesi & K.D. Hyde) Y. Marín & Crous	MFLUCC 13-0349 T	KP765682	KP765680	KP765681	NA
*Bambusaria bambusae* (J.N. Kapoor & H.S. Gill) Jaklitsch, D.Q. Dai, K.D. Hyde & Voglmayr	CBS 139763	KP687962	KP687813	KP687813	KP687983
*Bezerromyces brasiliensis* J.D.P. Bezerra, Souza-Motta & Crous	CBS 141545 T	KX518627	KX470390	KX518623	KX518631
*Bezerromyces pernambucoensis* J.D.P. Bezerra, C.M. Souza-Motta & Crous	CBS 141546 T	KX518628	KX470391	KX518624	KX518632
*Bezerromyces pseudobrasiliensis* Crous	CBS 141535	KX518630	KX470393	KX518626	KX518634
*Bezerromyces pseudobrasiliensis* Crous	CBS 141536 T	KX518629	KX470392	KX518625	KX518633
*Botryobambusa fusicoccum* Phookamsak, Jian K. Liu & K.D. Hyde	MFLUCC 11-0143 T	JX646826	NR_111793	JX646809	NA
*Botryosphaeria dothidea* (Moug.) Ces. & De Not.	CBS 115476 T	DQ677998	KF766151	DQ678051	DQ767637
*Chlamydosporoides sinensis* H. Pan & Zhi.Y. Zhang	CGMCC 3.29099 T	PV984083	PV984069	PV984076	PX000681
*Chlamydosporoides sinensis* H. Pan & Zhi.Y. Zhang	ZY 24.004	PV984084	PV984070	PV984077	PX000682
*Chlamydosporoides sinensis* H. Pan & Zhi.Y. Zhang	ZY 24.005	PV984085	PV984071	PV984078	PX000683
*Chlamydosporoides sinensis* H. Pan & Zhi.Y. Zhang	ZY 24.006	PV984086	PV984072	PV984079	PX000684
*Chlamydosporoides guizhouensis* H. Pan & Zhi.Y. Zhang	CGMCC 3.29100 T	PV984087	PV984073	PV984080	PX000685
*Chlamydosporoides guizhouensis* H. Pan & Zhi.Y. Zhang	ZY 24.008	PV984088	PV984074	PV984081	PX000686
*Chlamydosporoides guizhouensis* H. Pan & Zhi.Y. Zhang	ZY 24.009	PV984089	PV984075	PV984082	PX000687
*Cophinforma atrovirens* (Mehl & Slippers) A. Alves & A.J.L. Phillips	MFLUCC 11-0425	JX646833	JX646800	JX646817	NA
*Dematiopleospora mariae* Wanas., Camporesi, E.B.G. Jones & K.D. Hyde	MFLUCC 13-0612 T	KJ749652	KJ749654	KJ749653	KJ749655
*Excipulariopsis narsapurensis* (Subram.) Spooner & P.M. Kirk	KUMCC 21-0464	NA	OQ379007	OQ379418	OQ378997
*Excipulariopsis narsapurensis* (Subram.) Spooner & P.M. Kirk	KUMCC 21-0465	NA	OQ379008	OQ379419	OQ378998
*Excipulariopsis narsapurensis* (Subram.) Spooner & P.M. Kirk	NFCCI 5470	NA	OQ787041	NA	NA
*Glyphium elatum* (Grev.) H. Zogg	BPI 892669	KM220934	KM220943	KM220937	KM220932
*Glyphium elatum* (Grev.) H. Zogg	BPI 892671	KM220936	KM220945	KM220939	KM220933
*Helicangiospora lignicola* Boonmee, Bhat & K.D. Hyde	MFLUCC 11-0378	KF301539	KF301523	KF301531	KF301552
*Helicoma chiangraiense* (Boonmee & K.D. Hyde) Y.Z. Lu	MFLUCC 10-0115	JN865176	JN865200	JN865188	KF301551
*Helicoma fagacearum* (Boonmee & K.D. Hyde) Y.Z. Lu	MFLUCC 11-0379	KF301540	KF301524	KF301532	KF301553
*Heveicola xishuangbannaensis* R.F. Xu, K.D. Hyde & Tibpromma	KUMCC 21-0086 T	MZ824591	MZ803113	MZ803124	OL353340
*Heveicola xishuangbannaensis* R.F. Xu, K.D. Hyde & Tibpromma	KUMCC 21-0087	MZ824592	MZ803122	MZ803123	OL353341
*Hysteropatella clavispora* (Peck) Höhn.	CBS 247.34	DQ678006	NA	AY541493	DQ677901
*Myrmaecium fulvopruinatum* (Berk.) Jaklitsch & Voglmayr	CBS 139058 T	KP687968	KP687861	KP687861	KP688030
*Myrmaecium rubricosum* (Fr.) Fuckel	CBS 139068	KP687979	KP687885	KP687885	KP688053
*Parawiesneriomyces chiayiensis* Tennakoon, C.H. Kuo & K.D. Hyde	MFLUCC 20-0041 T	MW079359	MW063178	MW063239	NA
*Parawiesneriomyces chiayiensis* Tennakoon, C.H. Kuo & K.D. Hyde	NCYUCC 19-0065	MW079360	MW063179	MW063240	NA
*Parawiesneriomyces syzygii* Crous & M.J. Wingf.	CBS 141333 T	NA	KX228288	KX228339	NA
*Patellaria atrata* (Hedw.) Fr.	CBS 958.97	NA	NA	GU301855	GU349038
Patellaria cf. atrata (Hedw.) Fr.	BCC 28877	GU371837	NA	GU371829	NA
*Phaeotrichum benjaminii* Malloch & Cain	CBS 541.72 T	AY016348	NA	AY004340	DQ677892
*Phalangispora nawawii* Kuthub.	LAMIC 041712	NA	KR822207	KR869798	NA
*Phalangispora sinensis* J.S. Guo & Z.F. Yu	YMF 1.02677	MH031743	NA	MH031750	MH992087
*Phalangispora sinensis* J.S. Guo & Z.F. Yu	YMF 1.03656 T	MH031742	NA	MH031749	MH992086
*Phyllosticta ampelicida* (Engelm.) Aa	CBS 237.48	DQ678034	NA	DQ678085	NA
*Phyllosticta citricarpa* (McAlpine) Aa	CBS 102374	GU296151	FJ538313	GU301815	GU349053
*Populocrescentia forlicesenensis* Wanas., Camporesi, E.B.G. Jones & K.D. Hyde	MFLUCC 14-0651 T	KT306955	KT306948	KT306952	NA
*Pseudogliophragma indicum* Phadke & V.G. Rao	MTCC 11985 ET	KM052852	KM052850	KM052851	NA
*Setosynnema yunnanense* Y.L. Bai & Z.F. Yu	YMF 1.02199	MH031746	NA	MH031753	NA
*Setosynnema yunnanense* Y.L. Bai & Z.F. Yu	YMF 1.03964 T	MH031747	ON231810	MH031754	NA
*Speiropsis pedatospora* Tubaki	CBS 397.59 T	NA	MH857901	MH869443	NA
*Speiropsis scopiformis* Kuthub. & Nawawi	LAMIC 005111	NA	KR822206	KR869792	NA
*Trichodelitschia bisporula* Kuthub. & Nawawi	CBS 262.69	GU349000	NA	GU348996	GU349020
*Trichodelitschia munkii* N. Lundq.	Kruys201	DQ384070	NA	DQ384096	NA
*Tubeufia chiangmaiensis* Boonmee & K.D. Hyde	MFLUCC 11-0514	KF301543	KF301530	KF301538	KF301557
*Tubeufia javanica* Penz. & Sacc.	MFLUCC 12-0545	KJ880035	KJ880034	KJ880036	KJ880037
*Valsaria insitiva* (Tode) Ces. & De Not.	CBS 127882 T	KP687980	KP687886	KP687886	KP688054
*Valsaria lopadostomoides* Jaklitsch & Voglmayr	CBS 139062 T	KP687972	KP687868	KP687868	KP688037
*Valsaria rudis* (P. Karst. & Har.) Theiss. & Syd. ex Petr. & Syd.	CBS 139066 T	KP687976	KP687879	KP687879	KP688047
*Wiesneriomyces aquaticus* R.J. Xu, K.D. Hyde & Q. Zhao	HKAS 136210 T	PQ218211	PQ168235	PQ152621	NA
*Wiesneriomyces conjunctosporus* Kuthub. & Nawawi	BCC 18525	KJ425436	NA	KJ425450	NA
*Wiesneriomyces conjunctosporus* Kuthub. & Nawawi	BCC 20803	KJ425439	NA	KJ425453	NA
*Wiesneriomyces conjunctosporus* Kuthub. & Nawawi	BCC 4027	KJ425440	NA	KJ425449	NA
*Wiesneriomyces conjunctosporus* Kuthub. & Nawawi	BCC 40633	KJ425442	NA	KJ425455	NA
*Wiesneriomyces javanicus* Koord.	ICMP 13181	NA	OR543704	NA	NA
*Wiesneriomyces laurinus* (Tassi) P.M. Kirk	BCC 18609	KJ425443	NA	KJ425459	NA
Wiesneriomyces laurinus (Tassi) P.M. Kirk	BCC 2922	KJ425447	NA	KJ425456	NA
*Wiesneriomyces laurinus* (Tassi) P.M. Kirk	BCC 3922	KJ425448	NA	KJ425457	NA
*Wiesneriomyces laurinus* (Tassi) P.M. Kirk	BCC 40614	KJ425444	NA	KJ425460	NA
*Wiesneriomyces laurinus* (Tassi) P.M. Kirk	CBS 101058	NA	KR822217	KR869789	NA
*Wiesneriomyces laurinus* (Tassi) P.M. Kirk	CBS 101143	NA	KR822215	KR869790	NA
*Wiesneriomyces laurinus* (Tassi) P.M. Kirk	CBS 506.48	NA	KR822212	MH867994	NA
*Wiesneriomyces laurinus* (Tassi) P.M. Kirk	LAMIC 028912	NA	KR822208	KR869805	NA
*Wiesneriomyces laurinus* (Tassi) P.M. Kirk	LAMIC 036112	NA	KR822209	KR869804	NA
*Wiesneriomyces laurinus* (Tassi) P.M. Kirk	MFLU 2322	OR648180	OR659468	OR663920	NA
*Wiesneriomyces laurinus* (Tassi) P.M. Kirk	MFLUCC 17-0076	MN168759	MN168764	MN168761	MT050455
*Wiesneriomyces laurinus* (Tassi) P.M. Kirk	MFLUCC 19-0073	MW079357	MW063176	MW063237	NA
*Wiesneriomyces laurinus* (Tassi) P.M. Kirk	NCYUCC 19-0135	MW079358	MW063177	MW063238	NA
*Wiesneriomyces laurinus* (Tassi) P.M. Kirk	ZHKUCC 22-0008	OM780298	OM780284	OM780294	NA
*Wiesneriomyces laurinus* (Tassi) P.M. Kirk	ZHKUCC 22-0009	OM780306	OM780286	OM780295	NA
*Wiesneriomyces soli* Crous	CPC 47992 T	NA	PV664928	PV664955	PV664042
*Dendrographa decolorans* (Turner & Borrer ex Sm.) Ertz & Tehler	DUKE47570	AY548809	AY548808	AY548815	DQ883725
*Dendrographa minor* Darb.	Sparrius 7999 (BR)	AY548803	AY548802	AY548810	NA

Notes: T: Ex-type; ET: Ex-epitype; BCC: BIOTEC Culture Collection (Thailand); CBS: Centraalbureau voor Schimmelcultures (The Netherlands); CGMCC: China General Microbial Culture Collection Center (China); HKAS: Kunming Institute of Botany Academia Sinica (China); ICMP: International Collection of Microorganisms from Plants (New Zealand); KUMCC: Kunming Institute of Botany Culture Collection (China); MFLU: Mae Fah Luang University (Thailand); MFLUCC: Mae Fah Luang University Culture Collection (Thailand); NCYUCC: National Chiayi University Culture Collection (China); NFCCI: National Fungal Culture Collection of India (India); YMF: Key Laboratory of Industrial Microbiology and Fermentation Technology of Yunnan (China); ZHKUCC: University of Agriculture and Engineering Culture Collection (China); Other acronyms represent personal collections; NA, not available. DNA sequences for the new isolates are in bold.

### ﻿Phylogenetic analyses

Lasergene software (version 6.0, DNASTAR) was used to edit the ambiguous bases of the PCR amplicon sequences. From an initial BLAST search, it was shown that these new sequences belonged to Wiesneriomycetaceae. Therefore, sequences were obtained from recently published data (Bezerra et al. 2017; Guo et al. 2019; Xu et al. 2022; Yang et al. 2023). Sequences were aligned with MAFFT v7.037 (Katoh and Standley 2013) and alignments trimmed with MEGA v6.06 (Tamura et al. 2013). Combined sequences of SSU, ITS, LSU, and *tef1* were obtained using PhyloSuite v1.2.3 (Xiang et al. 2023). The most appropriate models of sequence evolution for Bayesian analysis (BI) and the maximum likelihood (ML) analysis by ModelFinder (Kalyaanamoorthy et al. 2017) in PhyloSuite v1.2.3 (Xiang et al. 2023) were used.

The Maximum Likelihood analysis (ML) was run in IQ-TREE v1.6.11 (Nguyen et al. 2015) with 10,000 bootstrap tests, using the ultrafast algorithm (Minh et al. 2013). The Bayesian analysis was conducted in MrBayes v.3.2 (Ronquist et al. 2012) in PhyloSuite v1.2.3 (Xiang et al. 2023). The Markov Chain Monte Carlo (MCMC) method was used to perform 5 × 10^7^ simulations with a sampling frequency of 10^3^ generations and a 25% burn-in. After the analysis was finished, Tracer v1.5 (Drummond and Rambaut 2007) was used to determine burn-in and confirm that both runs had converged. Multilocus phylogenetic trees were opened and checked using FigTree v 1.4.2 (Rambaut 2012), and the ﬁnal trees were edited in Microsoft PowerPoint by inserting statistical supports from ML and BI.

## ﻿Results

### ﻿Phylogenetic analyses

To determine the phylogenetic placement of the new isolates within Wiesneriomycetaceae, a dataset consisting of combined SSU, ITS, LSU, and *tef1* sequences was analyzed. *Dendrographa minor* Darb. (Sparrius 7999 BR) and *D. decolorans* (Turner & Borrer ex Sm.) Ertz & Tehler (DUKE 47570) were selected as outgroups. The final alignment included 79 taxa and consisted of 3,181 nucleotides positions (SSU, 964 bp; ITS, 516 bp; LSU, 831 bp; and *tef1*, 870 bp), including gaps. Maximum likelihood and Bayesian analyses were performed, respectively, and presented consistent topologies. Bayesian posterior probabilities were calculated with a final average standard deviation of split frequencies of less than 0.01.

**Figure 1.**
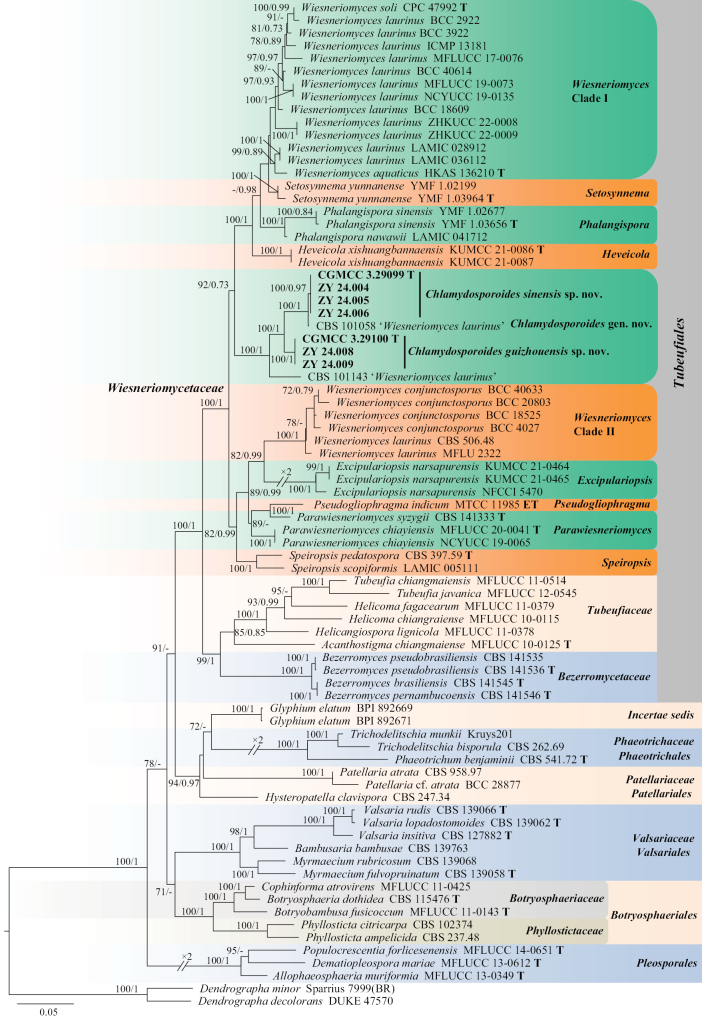
The maximum likelihood phylogenetic tree of *Chlamydosporoides* and related taxa based on concatenated sequences of SSU, ITS, LSU, and *tef1*, with *Dendrographa minor* (Sparrius 7999(BR)) and *D. decolorans* (DUKE 47570) as outgroups. Maximum likelihood bootstrap values (MLBV ≥ 70%) and Bayesian inference posterior probabilities (BIPP ≥ 0.7) are shown at the nodes, separated by slashes “/”. Strains obtained in this study are bold. Some branches are shortened for overall tree layout, indicated by “//”, with “×” denoting the fold of shortening.

The phylogenetic trees showed that Wiesneriomycetaceae formed a strongly supported clade (100/1), sister to Tubeufiaceae and Bezerromycetaceae within Tubeufiales. *Wiesneriomyces* was divided into two clades: Clade I comprised 14 isolates but had lower support, Clade II comprises six isolates and received extremely high support (100/1) (Fig. 1). *Excipulariopsis*, *Heveicola*, *Phalangispora*, *Setosynnema*, and *Speiropsis* each formed robust monophyletic clades. *Parawiesneriomyces* and *Pseudogliophragma* clustered together with moderate support (89/-). In addition, our new isolates (ZY 24.003–24.009) and ‘*Wiesneriomyces laurinus*’ (CBS 101058 and CBS 101143) formed a single, undescribed clade with high support (100/1, Fig. 1).

### ﻿Taxonomy

**Dothideomycetes O.E. Erikss. & Winka.**

**Tubeufiales Boonmee & K.D. Hyde**

**Wiesneriomycetaceae Suetrong, Rungjindamai, Somrithipol. & E.B.G. Jones**

#### ﻿ Chlamydosporoides

Taxon classificationFungiTubeufialesWiesneriomycetaceae

H. Pan & Zhi.Y. Zhang gen. nov.

89AF693F-3ED0-5412-96C6-D91D58F0179B

860151

##### Etymology.

Refers to its production of chlamydospore-like conidia.

##### Geographical distribution.

Guizhou Province, China.

##### Description.

***Hyphae*** branched, septate, hyaline to brown, smooth, sometimes hyphopodia-like structures creating a cauliflower-like appearance. ***Conidiogenous cells*** hyaline, smooth, solitary, cylindrical to clavate, straight or curved, aseptate. ***Conidia*** chlamydospore-like, solitary, hyaline to brown, non-septate, pyriform, globose to ellipsoid, or irregular shapes, sessile or borne on conidiogenous cells. ***Chlamydospores*** borne on hyphae, solitary, catenate, or grape-like, brown to dark brown. Setae, Conidiomata, and Sexual morph unknown.

**Type species.***Chlamydosporoides sinensis* H. Pan & Zhi.Y. Zhang.

**Notes.** Currently, the family Wiesneriomycetaceae comprises eight genera: *Excipulariopsis*, *Heveicola*, *Parawiesneriomyces*, *Phalangispora*, *Pseudogliophragma*, *Setosynnema*, *Speiropsis*, and *Wiesneriomyces* (Wijayawardene et al. 2020; Xu et al. 2022; Hyde et al. 2024). Molecular sequences for all genera are available in public databases (Table 1, Xu et al. 2022; Yang et al. 2023). Our multi-locus phylogenetic analysis revealed that, with the exception of *Wiesneriomyces* and *Parawiesneriomyces*, the remaining genera formed a strongly supported monophyletic clade (Fig. 1). The novel isolates obtained in this study, along with ‘*Wiesneriomyces laurinus*’ (CBS 101058 and CBS 101143), constituted a distinct subclade within Wiesneriomycetaceae (Fig. 1). Morphologically, no sexual morph has been reported in this family to date, while all known genera share the characteristic of producing conidia in acropetal chains (Xu et al. 2022). The newly proposed genus is primarily distinguished from other genera in Wiesneriomycetaceae by the absence of conidia produced in acropetal chains, setae, synnemata, sporodochium or stroma.

#### ﻿ Chlamydosporoides sinensis

Taxon classificationFungiTubeufialesWiesneriomycetaceae

H. Pan & Zhi.Y. Zhang sp. nov.

F549634B-57FF-531F-90F4-6A80A0B69D59

860152

Fig. 2

##### Etymology.

The epithet “*sinensis*” (Lat.) refers to China, where the species was collected.

##### Type.

China: • Guizhou Province, Guiyang City, Huaxi District, Guizhou Minzu University, 26°46'25"N, 106°66'95"E, from epiphytic soil of *Camphora officinarum* Boerh.ex Fabr., 25 July 2024, Heng Pan and Zhi-Yuan Zhang (holotype HMAS 354106, dried culture; ex-type CGMCC 3.29099 = ZY 24.003; *ibid.* ZY 24.004).

**Figure 2.**
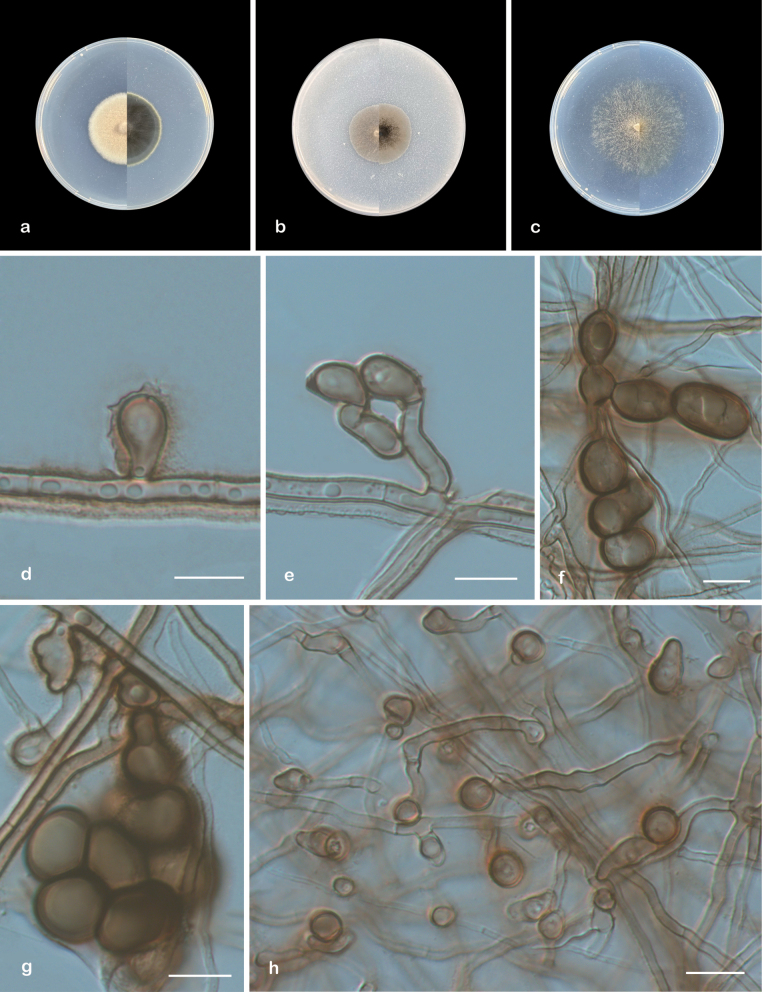
*Chlamydosporoides sinensis* (ex-type CGMCC 3.29099). a–c. Upper and reverse views of cultures on PDA, OA, and SNA after 14 days at 25 °C; d, h. Conidia e. Conidia and conidiogenous cell; f, g. Chlamydospores. Scale bars: 10 µm (d–h).

##### Description.

***Culture characteristics*** (14 days at 25 °C): Colony on PDA 39–43 mm diam. flat, agate gray to platinum gray from center to margin, nearly circular, margin regular; reverse: light ivory to oyster white from center to margin. Colony on OA 30–34 mm diam. flat, graphite gray to green beige from center to margin, nearly circular, margin regular; reverse: dark ivory. Colony on SNA 53–58 mm diam. fluffy, hyphae sparse, honey yellow; reverse: honey yellow.

***Hyphae*** branched, septate, hyaline to brown, smooth, 1–4 μm wide. sometimes hyphopodia-like structures creating a cauliflower-like appearance. ***Conidiogenous cells*** smooth, solitary, cylindrical to clavate, straight or curved, aseptate, 13–15.5 × 2.5–3.5 μm. ***Conidia*** chlamydospores-like, solitary, hyaline to brown, smooth, non-septate, pyriform, globose to ellipsoid, or irregular shapes, sessile or borne on conidiogenous cells, 6–12 × 5–7 μm (av. 8.2 × 6.1, n = 50). ***Chlamydospores*** borne on hyphae, solitary, catenate, or grape-like, brown to dark brown, 11–17.5 × 7.5–13 μm (av. 12.8 × 10, n = 30). Setae, Conidiomata, and Sexual morph unknown.

##### Geographical distribution.

Guizhou Province, China.

##### Additional material examined.

China: • Guizhou Province, Guiyang City, Huaxi District, Guizhou Minzu University, 26°46'15"N, 106°66'81"E, from epiphytic soil of *Camphora officinarum*, 25 July 2024, Heng Pan and Zhi-Yuan Zhang ZY 24.005, *ibid.* ZY 24.006).

##### Notes.

Phylogenetic analysis revealed that four new isolates (ZY 24.003–24.006) formed a distinct subclade within *Chlamydosporoides* with strong statistical support (100/0.97, Fig. 1). Morphologically, *Chlamydosporoides sinensis* differs from *C. guizhouensis* by its smooth-walled conidia and the production of abundant chlamydospores. Furthermore, these species exhibit significant molecular differentiation, as evidenced by sequence comparisons of three loci: *C. sinensis* (ex-type CGMCC 3.29099) shows 91.6% ITS (550/600 bp, 13 gaps), 98.3% LSU (839/853 bp, one gap), and 95.9% *tef1* (892/930 bp, one gap) similarity when compared with *C. guizhouensis* (ex-type CGMCC 3.29100). Due to the lack of original descriptions for strains CBS 101058 and CBS 101143 (designated as ‘*W. laurinus*’), we restricted our comparison to sequence analyses. CBS 101058 demonstrated 97.6% ITS (527/540 bp, 2 gaps) and 100% LSU (834/834 bp, no gaps) similarity with *C. sinensis* (CGMCC 3.29099), suggesting potential conspecificity. In contrast, CBS 101143 showed markedly lower similarity (76.3% ITS [449/588 bp, 51 gaps] and 97.8% LSU [816/834 bp, one gap]) relative to the *C. sinensis* ex-type strain.

#### ﻿ Chlamydosporoides guizhouensis

Taxon classificationFungiTubeufialesWiesneriomycetaceae

H. Pan & Zhi.Y. Zhang sp. nov.

7D02A25C-6780-5336-9CF6-9E534A405218

860154

Fig. 3

##### Etymology.

The epithet “*guizhouensis*” (Lat.) refers to Guizhou Province, where the species was collected.

**Figure 3.**
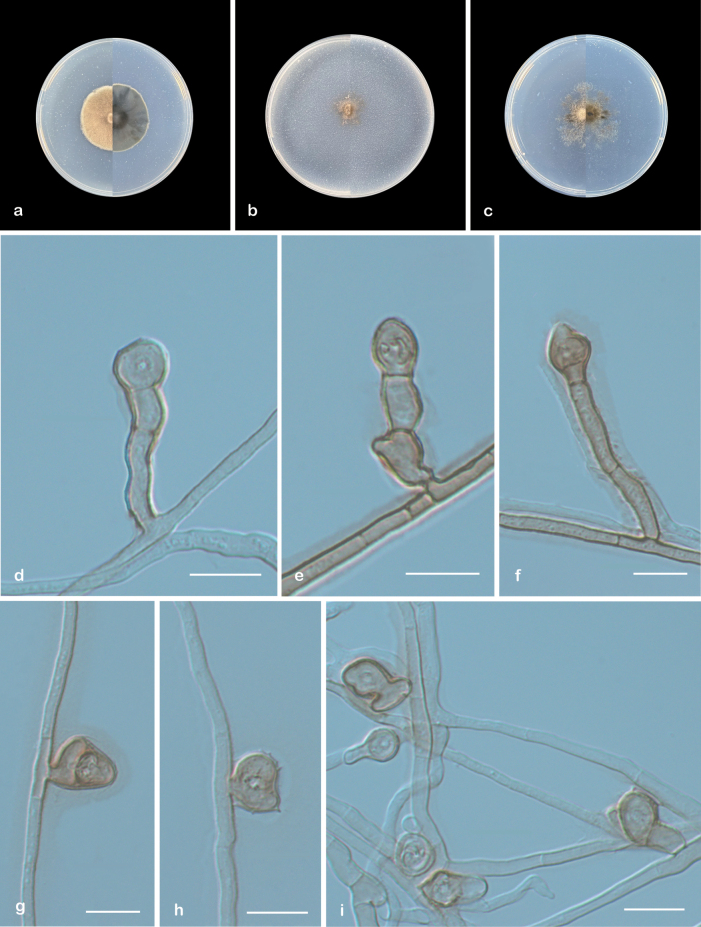
*Chlamydosporoides guizhouensis* (ex-type CGMCC 3.29100). a–c. Upper and reverse views of cultures on PDA, OA, and SNA after 14 days at 25 °C; d–f. Conidia and conidiogenous cell; g–i. Conidia. Scale bars: 10 µm (d–i).

##### Type.

China: • Guizhou Province, Guiyang City, Huaxi District, South Campus of Guizhou University, 26°42'23"N, 106°67'17"E, from epiphytic soil of *Catalpa ovata* G. Don, 28 September 2024, Heng Pan (holotype HMAS 354107, dried culture; ex-type CGMCC 3.29100 = ZY 24.007; *ibid.* ZY 24.008).

##### Description.

***Culture characteristics*** (14 days at 25 °C): Colony on PDA 31–39 mm diam. flat, gray white to platinum gray from center to margin, nearly circular, margin regular; reverse: light ivory to oyster white from center to margin. Colony on OA 21–25 mm diam. flat, brown beige to dark ivory from center to margin, margin irregular; reverse: green beige. Colony on SNA 33–39 mm diam. fluffy, honey yellow to white from center to margin, hyphae sparse; reverse: white.

***Hyphae*** branched, septate, hyaline to brown, smooth, 1–3 μm wide. hyphopodia-like structures creating a cauliflower-like appearance. ***Conidiogenous cells*** hyaline, smooth, solitary, cylindrical to clavate, straight or curved, aseptate, 7.5–12.5 (–18) × 3–6 μm. ***Conidia*** chlamydospores-like, solitary, hyaline to brown, rough, non-septate, pyriform, globose to ellipsoid, or irregular shapes, sessile or borne on conidiogenous cells, 5.5–11.5 × 6–9.5 μm (av. 8.6 × 7.4, n = 50). Setae, Conidiomata, Chlamydospores, and Sexual morph unknown.

##### Geographical distribution.

Guizhou Province, China.

##### Additional material examined.

China: • Guizhou Province, Guiyang City, Huaxi District, South Campus of Guizhou University, 26°42'35"N, 106°67'20"E, from epiphytic soil of *Catalpa ovata*, 28 September 2024, Heng Pan (ZY 24.009).

##### Notes.

Phylogenetic analysis revealed that four new isolates (ZY 24.007–24.009) formed a distinct subclade with strong statistical support (100/1). Morphologically, for differences between *Chlamydosporoides guizhouensis* and *C. sinensis*, see the notes on *C. sinensis* above. Furthermore, in a comparison of ITS and LSU nucleotides, CBS 101143 has 79.2% and 98.5% similarity in ITS (466/588 bp, 48 gaps) and LSU (821/833 bp, one gap), which is different from *C. guizhouensis* (CGMCC 3.29100).

### ﻿Key to genera in *Wiesneriomycetaceae* (Revised from Xu et al. 2022)

**Table:** 

1	Synnemata, sporodochium or stroma present	**2**
–	Synnemata, sporodochium or stroma absent	*** Chlamydosporoides ***
2	With setae at the edge of the sporodochium or basal stroma	**3**
–	Without setae at the base of synnemata, sporodochium or stroma	4
3	Conidia hyaline or brown to dark brown, unbranched	5
–	Conidia pale-brown, toruliform, with two to three branches	*** Phalangispora ***
4	Conidia hyaline	**6**
–	Conidia coloured	**7**
5	Conidia subcylindrical to cylindrical	**8**
–	Conidia fusiform	*** Excipulariopsis ***
6	Conidia aggregated into heads, narrowly fusiform to lanceolate, septate	*** Pseudogliophragma ***
–	Conidia aggregated in heads, filiform, with an isthmus at the central septum tapered towards each end	*** Setosynnema ***
7	Conidia pale to mid-brown, with three to five radiate arms	*** Speiropsis ***
–	Conidia brown, 1–4 to multiseptate, with dark brown band at the septa	*** Heveicola ***
8	Conidia cylindrical connected by short isthmi separating each	*** Wiesneriomyces ***
–	Conidia subcylindrical joined by a narrow isthmus	*** Parawiesneriomyces ***

## ﻿Discussion

Members of Wiesneriomycetaceae are mainly found in tropical and subtropical regions on decaying leaves, wood, and soil (Suetrong et al. 2014; Pratibha et al. 2015; Crous et al. 2016; Crous 2025). Based on molecular and morphological data, a novel genus, *Chlamydosporoides*, along with two new species, were isolated from tree hole soils in Guizhou Province. Phylogenetic analyses provided strong and robust evidence for these new fungal classifications. Current phylogenetic analyses of Wiesneriomycetaceae have been extensively conducted using *tef1*, ITS, LSU, *rpb2*, and SSU gene regions, yielding robust results (Suetrong et al. 2014; Bezerra et al. 2017; Tennakoon et al. 2021; Xu et al. 2022; Crous 2025). This study used concatenated SSU, ITS, LSU, and *tef1* sequences, consistent with Bezerra et al. (2017).

*Chlamydosporoides*, *Excipulariopsis*, *Heveicola*, *Phalangispora*, *Setosynnema*, and *Speiropsis* each formed robust monophyletic clades (Fig. 1). *Excipulariopsis* was established with *E. narsapurensis* as the type species (Spooner and Kirk 1982), and the asexual morph has raised stroma, cylindrical, hyaline, holoblastic conidiogenous cells and fusiform, transversely multi-septate, brown, verruculose, acrogenous conidia with a truncate base (Spooner and Kirk 1982; Pem et al. 2019). Yang et al. (2023) collected a specimen (HKAS 122680) from decaying bark of *Mangifera indica* L. in Menglong Village, Honghe Prefecture, Yunnan Province, China, with living cultures deposited as KUMCC 21-0464 and KUMCC 21-0465. The morphological characteristics of HKAS 122680 resembled *E. narsapurensis* (BISH 594584), leading to its identification as this species. Phylogenetic analyses based on molecular sequences obtained from KUMCC 21-0464 and KUMCC 21-0465 placed *Excipulariopsis* within Tubeufiaceae, though the analysis did not include molecular data from any taxa of Wiesneriomycetaceae (Yang et al. 2023). Our phylogenetic sampling encompassed three families (Bezerromycetaceae, Tubeufiaceae, and Wiesneriomycetaceae) within Tubeufiales, revealing that *Excipulariopsis* forms a distinct subclade in Wiesneriomycetaceae with strong statistical support (Fig. 1). Therefore, *Excipulariopsis* should be classified under Wiesneriomycetaceae.

*Pseudogliophragma indicum* Phadke & V.G. Rao was described from decaying twigs of *Mangifera indica* L. in India (Phadke and Rao 1980). Pratibha et al. (2015) obtained a specimen (HCIO 51503) from unidentified decayed twigs and designated it as the epitype of *P. indicum*, with the ex-epitype culture deposited as MTCC 11985. Crous et al. (2016) introduced *Parawiesneriomyces* with *P. syzygii* Crous & M.J. Wingf. as the type species, which was collected from leaves of *Syzygium jambos* (L.) Alston (Myrtaceae) in France. Subsequently, Tennakoon et al. (2021) described an additional new species in the genus, *P. chiayiensis*. In the phylogenetic tree, *P. syzygii* (ex-type CBS 141333) and *Pseudogliophragma indicum* (ex-epitype MTCC 11985) formed a highly supported subclade (100/1, Fig. 1), which was distinct from *Parawiesneriomyces chiayiensis*. However, the clustering of *Parawiesneriomyces* and *Pseudogliophragma* received only moderate statistical support (89/-, Fig. 1). Therefore, further research is needed to elucidate the relationship among these two genera.

*Wiesneriomyces* was divided into two clades: Clade I comprised *W. soli* (ex-type CPC 47992), *W. aquaticus* (ex-type HKAS 136210), and 12 isolates identified as *W. laurinus*, although this clade received only weak statistical support (Fig. 1). Clade II consisted of four *W. conjunctosporus* isolates and two *W. laurinus* isolates, with this clade receiving strong statistical support (100/1). Currently, phylogenetic analysis has become one of the essential approaches in fungal taxonomy, and “splitting” appears to be the prevailing trend in contemporary fungal systematics (Yang 2020). However, the same species subjected to different environmental pressures across habitats should inherently exhibit certain degrees of genetic variability. Sklenář et al. (2022) conducted a comprehensive integrative taxonomic analysis of *Aspergillus* series *Versicolores* using a large dataset comprising 518 strains, ultimately reducing the number of recognized species in this series from 17 to 4. Therefore, Clade I, *Setosynnema*, *Phalangispora*, and *Heveicola* likely represent a single genus, while Clade II probably constitutes a new genus within Wiesneriomycetaceae, though these hypotheses require verification through more extensive datasets and comprehensive analyses.

## Supplementary Material

XML Treatment for Chlamydosporoides

XML Treatment for Chlamydosporoides sinensis

XML Treatment for Chlamydosporoides guizhouensis

## ﻿Additional information

### Use of AI

No use of AI was reported.

### Author ORCIDs

Heng Pan https://orcid.org/0009-0003-7472-3199

Kai-Rong Wang https://orcid.org/0009-0000-6866-4047

Ming-Yi Zhang https://orcid.org/0009-0000-0691-3941

Xiao-Kang Ren https://orcid.org/0009-0003-1278-9352

Bing-Da Sun https://orcid.org/0000-0003-0272-6422

Gang Tao https://orcid.org/0000-0002-0882-3752

Zhi-Yuan Zhang https://orcid.org/0000-0003-2031-7518

### Data availability

All of the data that support the findings of this study are available in the main text.
